# Designing a Patient Portal for Patient-Centered Care: Cross-Sectional Survey

**DOI:** 10.2196/jmir.9497

**Published:** 2018-10-01

**Authors:** Steve Alfons Van den Bulck, Rosella Hermens, Karin Slegers, Bert Vandenberghe, Geert Goderis, Patrik Vankrunkelsven

**Affiliations:** 1 Academic Center for General Practice Department of Public Health and Primary Care KU Leuven Leuven Belgium; 2 Scientific Institute for Quality in Healthcare, Radboud Institute for Health Sciences Radboud University Medical Center Radboud University Nijmegen Nijmegen Netherlands; 3 Meaningful Interactions Lab Imec KU Leuven Leuven Belgium

**Keywords:** cross-sectional studies, consumer health informatics, electronic health record, patient portal, personal health records

## Abstract

**Background:**

In recent literature, patient portals are considered as important tools for the delivery of patient-centered care. To date, it is not clear how patients would conceptualize a patient portal and which health information needs they have when doing so.

**Objective:**

This study aimed (1) to investigate health information needs, expectations, and attitudes toward a patient portal and (2) to assess whether determinants, such as patient characteristics, health literacy, and empowerment status, can predict two different variables, namely the importance people attribute to obtaining health information when using a patient portal and the expectations concerning personal health care when using a patient portal.

**Methods:**

We conducted a cross-sectional survey of the Flemish population on what patients prefer to know about their digital health data and their expectations and attitudes toward using a patient portal to access their electronic health record. People were invited to participate in the survey through newsletters, social media, and magazines. We used a questionnaire including demographics, health characteristics, health literacy, patient empowerment, and patient portal characteristics.

**Results:**

We received 433 completed surveys. The health information needs included features such as being notified when one’s health changes (371/396, 93.7%), being notified when physical parameters increase to dangerous levels (370/395, 93.7%), observing connections between one’s symptoms or diseases or biological parameters (339/398, 85.2%), viewing the evolution of one’s health in function of time (333/394, 84.5%), and viewing information about the expected effect of treatment (349/395, 88.4%). Almost 90% (369/412) of respondents were interested in using a patient portal. Determinants of patients’ attachment for obtaining health information on a patient portal were (1) age between 45 and 54 years (*P*=.05); (2) neutral (*P*=.03) or interested attitude (*P*=.008) toward shared decision making; and (3) commitment to question physicians’ decisions (*P*=.03, *R^2^*=0.122). Determinants of patients’ expectations on improved health care by accessing a patient portal were (1) lower education level (*P*=.04); (2) neutral (*P*=.03) or interested attitude (*P*=.008) toward shared decision making; and (3) problems in understanding health information (*P*=.04; *R^2^*=0.106).

**Conclusions:**

The interest in using a patient portal is considerable in Flanders. People would like to receive alerts or some form of communication from a patient portal in case they need to act to manage their health. Determinants such as education, attached importance to shared decision making, difficulties in finding relevant health information, and the attached importance in questioning the decisions of physicians need to be considered in the design of a patient portal.

## Introduction

The expression “no decision about me, without me,” as formulated by the British National Health Service, emphasizes the importance of patient-centered care and shared decision making [1]. Patient-centered care, which is an important feature of high-quality health care, is defined as “providing care that is respectful of and responsive to individual patient preferences, needs, and values, and ensuring that patient values guide all clinical decisions” [2]. The Institute of Medicine considers patient-centered care as one of the 6 objectives to be achieved to improve the quality of US health care [2]. It can be promoted with the help of patient-centered health information technologies [3], such as a patient portal.

A patient portal is known to improve the quality of and access to health care [4]. In addition, a patient portal exerts a positive influence on health care utilization [5]. A patient portal can be defined as “An electronic application through which individuals can access, manage and share their health information, and that of others for whom they are authorized, in a private, secure, and confidential environment” [6,7]. More specifically, a patient portal can be described as an application that is linked (tethered) to the electronic health record of the doctor [8,9]. The functions of a patient portal often include a medication list, test results, a list of allergies, a problem list, social history, major illness, lifestyle, family history, and links to personalized information [7,9].

Although patient portals are considered important tools for the development of patient-centered care, the current use is not optimal, and portals are still less patient-centered than they could be [10-12]. Known barriers to the use of portals for patients and providers include security and privacy issues, the negative impact on the workflow and limited user-friendliness [4,13]. Benefits associated with the use of a patient portal are increased convenience and satisfaction for patients [14,15].

Patient safety could be improved by identifying errors in medication lists [16,17]. Moreover, providing Web access could lower the threshold for the patient-clinician contact [13]. In addition, the quality of care can be improved by the sustained use of some features of a patient portal. For example, Web-based prescription refills and secure messaging have an impact on some physiological measures important for the management of type 2 diabetes [18].

Patients’ interest and ability to use a patient portal are influenced by age, health literacy, and level of education. Patients are more likely to adopt a patient portal if its features align with their information needs and with the functionalities they require [19].

To date, however, it remains unclear what patients’ information needs and functionality requirements exactly are. In addition, participants in this study had no prior experience with patient portals as they do not exist in Belgium. The survey intro was the first exposure people had to the concept of a patient portal. Therefore, this study aimed to investigate patients’ information needs with regard to the concept of a patient portal and its presumed use to access their health data. Furthermore, this study aims to assess patients’ expectations and attitudes regarding a patient portal and its use to access electronic health records in primary care. Finally, we investigate whether patients’ information needs, expectations, and attitudes regarding a patient portal correlate with patient-related determinants such as personal characteristics, health literacy, and empowerment status.

## Methods

### Study Design

We conducted a cross-sectional survey on the digital health information needs of patients and their expectations and attitudes toward using a patient portal to access their electronic health record in primary care among inhabitants of the Dutch-speaking part of Belgium (Flanders).

### Study Population and Sample Size

The study population included inhabitants of the Flemish part of Belgium. For precise estimation of our results, we calculated the sample size using the free Web-based software Raosoft [20]. For a population size of 6,471,996, with a 5% margin of error and a 50% response distribution, we needed 385 completed surveys.

### Design and Domains of the Survey

Our open and voluntary survey was a convenience sample and consisted of 2 parts (Multimedia Appendix 1 for the English version and Multimedia Appendix 2 for the Dutch Version).

The first part was based on the Health Information Technology Evaluation Collaborative (HITEC) Consumer Health Information Technology Survey as designed by Patel et al [21]. The second part was based on findings from previous qualitative research. The first page of the survey contained the informed consent and a mandatory checkbox for participants to acknowledge that they have read and agree with the informed consent. The final page of the survey contained a mandatory checkbox for participants to acknowledge they wanted to send the information to the researchers. If not, the data were not stored. Multimedia Appendix 3 provides a detailed description of the survey methodology according to the Checklist for Reporting Results of Internet E-Surveys [22].

#### Part 1 of the Survey

The HITEC Consumer Survey, developed in the United States, aimed to characterize consumer attitudes toward personal health records and included questions on potential personal health record use, preferences, and attitudes toward personal health records, the use of computers and the internet, experience with health care, health characteristics, and demographics [21].

One researcher translated the HITEC Consumer Survey from English to Dutch, and 2 researchers with adequate English proficiency translated the Dutch survey independently from each other back to English to reduce language bias to a minimum [23,24]. Small differences in the translation were solved by consensus. In the Dutch translation, a patient portal was defined as “an electronic online tool to view and manage patients’ health care information stored in the electronic health record of their general physician” [8]. Of note, 2 questions were not included in the Dutch translation of the questionnaire—one about payment options and one about private insurance companies—because these were not applicable to the Belgian situation. A few other questions were slightly changed because of the different demographic situation in Belgium compared with that in the United States. For example, questions inquiring about ethnicity were reformulated to match the common Dutch terminology. Instead, of asking “what is your race” or “are you of Latino or Hispanic origin or descent,” we asked, “What is your mother’s, father’s, and your country of birth?”

#### Part 2 of the Survey

The questions in this part of the survey were based on the findings of a previous qualitative study (Multimedia Appendix 4). This study aimed to understand what motivated people to search for health information. For this purpose, cultural probes and the Lillidots method were used [25]. Cultural probes are objects, such as a diary or camera, provided to participants to self-report data; cultural probes perform similar to astronomic probes because they are also left behind and return with fragmentary data [26,27]. In the study mentioned above, cultural probes were used to sensitize participants about digital health data to make it easier for them to express their feelings and experiences regarding health information. Participants were then interviewed to learn what health topics they wanted to know more about and what their questions and expectations were concerning these topics. For this purpose, an ideation method, called the Lillidots method [25], was combined with a strategy to anthropomorphize the technology at hand (digital health data in this case). This method, detailed elsewhere [28], resulted in insights about the type of health data people are interested in, questions they have in this respect, and their motivations to ask these questions. The second part of the survey was based on these motivations and included questions such as “In your relationship with your doctor, how important is it for you to be able to question their decision” and “Please indicate how important each of the following aspects would be to you, if you had access to all the data in your patient portal.”

In a nutshell, the second part of the survey was developed based on the themes that emerged from the previous qualitative study: (1) health awareness; (2) coping; (3) effective care; (4) empowerment; (5) good health; (6) patient rights; and (7) getting recognition. These themes grouped several items, such as people want to understand whether and when care is necessary and compare possible treatments (effective care) or what reimbursements they are entitled to (patient rights). To validate these findings with a larger population a questionnaire was constructed based on these items (part 2 of the survey). Combining part 1 and 2 allowed us to use a quantitative research instrument with our bottom-up qualitative research method.

#### Pilot

A pilot was performed in a small sample of 6 Dutch-speaking Belgians to establish whether the survey was feasible, particularly regarding the ease of use and time of completion. All participants appraised the survey as user-friendly and completed it in an average time of 20 minutes, which was considered acceptable. We did not include any pilot data in the large sample for analysis.

### Data Collection

The data were collected between March 25 and September 1, 2016. Inhabitants of the Flemish part of Belgium were invited to participate through several channels—the member magazine of a health insurance with a print run expanding up to 1,200,000 copies, only this channel gave readers an opportunity to choose between Web-based reply and paper; the Web-based newsletter of the same health insurance; the newsletter of a website called “Health and Science,” which is an independent and evidence-based website to inform patients about health-related topics (12,000 subscribers); and the website of a well-known Flemish weekly magazine (knack.be), which receives 54,769 unique visitors and 141,304 (2.58 per visitor) page views per day [29] and their social media channels (Facebook and Twitter). In addition, some students of the Faculty of Social Sciences used their social networks to recruit participants. Two reminders were sent by the health insurance and the “Health and Science” newsletter to obtain the greatest possible response. People without access to a computer or the internet could phone to request a paper copy of the survey. This ensured involving as many people as possible and having a representative sample of the population to minimize selection bias. The paper surveys we received were manually entered into the database containing the results from the Web-based surveys.

### Analyses

#### Descriptive Statistics (Frequencies)

In this study, descriptive statistics (frequencies) were used to assess the following:

- Demographics (age, gender, mother tongue, employment status, education, income, internet use, and residence),
- Health characteristics (answers to questions on self-rated health status, the presence of a chronic disease, >3 annual visits to the primary care physician, and prescribed medication use),
- Health literacy (answers to questions on difficulties with finding relevant and reliable health information),
- Patient empowerment (answers to questions on the quality of health care received in the last 5 years and questions about the importance of shared decision making and being able to question physicians’ decisions),
- The potential use of a patient portal, attitude and expectations when using a patient portal (answers to questions on one’s interest in using a patient portal, types of information expected to be found in a patient portal, Web-based activities concerning health, frequency of using a patient portal, perceived usefulness of a patient portal, difficulty with using a patient portal, granting access to one’s own patient portal, and importance of certain features of a patient portal).

#### Determinant Analyses

To investigate whether determinants such as patient characteristics, health literacy, and patient empowerment predict the importance people attach to obtaining health information when accessing a patient portal and the expectations and attitudes of people toward the use of a patient portal, we used linear and logistic regression models. First, bivariate regression models were used to investigate associations between all variables. Only predictor variables that were significantly associated (*P* ≤.10) with the dependent variable in bivariate analyses were used for the multivariate analyses.

Bivariate and multivariate linear regression models were used to predict 2 different dependent variables. For one of these “the importance people attach to obtaining health information when using a patient portal,” we used the mean sum score of 5-point Likert scale answers (ranging from 1 “very important” to 5 “very unimportant”) to 12 questions asking about the importance of doing certain things with the help of a patient portal (Cronbach alpha=.897). We considered the mean sum score as an interval variable and used it as a dependent variable in a linear regression model. [30]

For the second dependent variable “expectations concerning one’s personal health care when using a patient portal,” we used the mean sum score of answers to 9 questions asking for the level of expected improvement on different aspects of health care when using a patient portal (ranging from 1 “will greatly improve” to 5 “will greatly worsen”; Cronbach alpha=.871). We used the mean sum score to compensate for missing data in ≥1 of the questions. Preliminary analyses were performed to ensure there was no violation of the assumption of normality, linearity, and multicollinearity. If a correlation (>.6) between independent variables was detected, only the variable with the greatest influence on the *R*^2^of the model was included in the multivariate analyses.

We used bivariate and multivariate logistic regression models to predict the attitude toward using a patient portal. We used answers to the question asking for the interest in using a patient portal as the dependent variable. This ordinal variable with 5 categories was recoded into a dichotomous variable (interested and not interested or neutral)

As predictor variables, we used answers to the questions asking for patient characteristics (age, employment status, family income, health status, education, and gender), patient empowerment (shared decision making, questioning the decisions of physicians, satisfaction with health care received the last 5 years), and health literacy (finding relevant information about health, evaluating the reliability of health information, and problems in understanding health information; Multimedia Appendix 5).

Except for the internal consistency (Cronbach alpha) of our composite dependent variables, we did not include any psychometric measures for our predictor variables because of the gap in the availability of sound psychometric measures for evaluating patient-facing eHealth technologies [31]. All statistical analyses were performed with IBM SPSS Statistics version 24 for Windows (IBM Corp, Armonk, NY, USA).

### Missing Data

To calculate the frequencies, missing data were excluded, and percentages were based on the number of nonmissing values. For the regression models, we used independent variables that had a low percentage of missing data (<15%). If values of any of the independent variables included missing data, the entire case was excluded for the analysis.

### Ethical Approval

This study was approved by the Social and Societal Ethics Committee of the Faculty of Social Sciences of KU Leuven on July 14, 2015 (first part of the survey) and on September 24, 2015 (second part of the survey) with the grant number G-2015 07 272.

## Results

### Demographics and Health Characteristics

The survey was completed by 433 people. While 10 surveys were submitted on paper, 423 were completed through the Web (Table 1). The completion rate of the Web-based survey was 91% (Multimedia Appendix 3). Differences between the 10 surveys submitted on paper and those completed on the Web were mainly age of respondents, age-related properties (eg, employment status), and their internet use. The mean age in the group of the paper surveys (n=10) was 68.6 years (SD 10.146), while it was 53 years (SD 16.497) in the Web-based group (n=423). All respondents of the paper surveys group were not working anymore (9/10 retired and 1/10 disabled), and 40% (4/10) did rarely or never used the internet, while only 0.2% (1/423) of the Web-based group rarely or never used the internet. The mean age of all the respondents (n=433) was 53.28 (SD 16.451) years.

Almost 92% (397/432) of participants reported that they were in excellent or good health, although 49.4% (213/431) reported they had a chronic disease, and 69.4% (300/432) were taking prescribed medication (Table 1).

### Health Literacy and Patient Empowerment

Finding relevant health information was considered difficult by 26.2% (106/404) of respondents, and assessing the reliability of the health care information found was considered difficult by 48.3% (195/404). More than 93% (377/404) of participants reported that they found shared decision making important, and 89.9% (363/404) of respondents thought it was important to be able to question the decisions made by physicians (Table 2).

**Table 1:** Demographics and health characteristics of the participants (N=433).

Characteristics		Participants, n (%)^a^
Male		187 (47.1)
Dutch-speaking		394 (99.0)
**Age (years)**		
	18-34	72 (19.1)
	35-44	39 (10.4)
	45-54	53 (14.1)
	55-64	95 (25.3)
	>65	117 (31.1)
**Employment status**		
	Employed	164 (41.3)
	Student	21 (5.3)
	Unemployed	51 (12.8)
	Retired	161 (40.6)
**Education**		
	Secondary school	130 (32.7)
	Bachelor degree	159 (40.1)
	Master degree or higher	108 (27.2)
**Family income**		
	€ <30,000	114 (28.9)
	€ 30,000-60,000	100 (25.3)
	€ >60,000	58 (14.7)
	Does not know or prefers not to disclose	123 (31.1)
**Internet use**		
	Internet use ≥1 time per day	423 (97.9)
	Searched information on health or disease on the Web	406 (94.4)
**Residence description**		
	Rural	202 (50.6)
	Urban	197 (49.4)
**Self-rated health status**		
	Excellent or very good	121 (28.0)
	Good or fair	276 (63.9)
	Poor	35 (8.1)
Chronic medical condition		213 (49.4)
Visited primary caregiver >3 times in a year		214 (50.1)
Taking prescribed medication		300 (69.4)

^a^Numbers may not sum to totals because of missing data. Percentages were calculated considering the missing data.

**Table 2:** Health literacy and patient empowerment (N=433).

Health literacy and patient empowerment		Participants, n (%)^a^
**Satisfaction with the quality of health care received in the last 5 years**		
	Satisfied	366 (84.5)
	Neutral	39 (9.0)
	Dissatisfied	28 (6.5)
**Finding relevant health information**		
	Difficult	106 (26.2)
	Not easy or not difficult	149 (36.9)
	Easy	149 (36.9)
**Assessing the reliability of Web-based health care information**		
	Difficult	195 (48.3)
	Not easy or not difficult	116 (28.7)
	Easy	93 (23.0)
**Importance of shared decision making**		
	Important	377 (93.3)
	Neutral	18 (4.5)
	Not important	9 (2.2)
**Importance of being able to questions physicians’ decisions**		
	Important	363 (89.9)
	Neutral	37 (9.1)
	Not important	4 (1.0)

^a^Numbers may not sum to totals because of missing data. Percentages were calculated considering the missing data.

### Patient Portal’s Potential Impact and Features

Most respondents were interested in the potential use of a patient portal (369/412, 89.6%; Table 3). The information that most people wanted to see in their patient portal were test results (381/410, 92.9%), current medication (345/410, 84.1%), immunization records (338/410, 82.4%), and their past medical visits, procedures, and surgeries (338/410, 82.4%). This corresponds with the Web-based health-related activities people are most interested in, namely viewing their medical records, test results, medication list (384/405, 94.8%); requesting appointments, referrals, and prescription refills (376/403, 93.3%); and signing up for reminders for preventive medicine (360/399, 90.2%).

Respondents were less interested in seeing their lifestyle choices (138/410, 33.7%) and information from devices to help monitor their health (199/410, 48.5%; Table 3).

The perceived impact of patient portal use varied. Only 22.3% (90/404) of respondents believed that the patient portal use would improve the security and privacy of their medical data, and 47.4% (192/405) of participants thought that using a patient portal would reduce the overall cost of their health care.

The majority (391/414, 94.4%) would give their primary care doctor permission to view information in their patient portal. The potential features of a patient portal that were considered important by respondents were being notified when certain physical parameters evolve toward dangerous levels (370/395, 93.7%), being notified when their health changes (371/396, 93.7%), being able to view the expected impact of treatment on personal health (349/395, 88.4%), being able to see connections between symptoms, disease(s), biological parameters, etc (339/398, 85.2%), and being able to view the evolution of their health in function of time (333/394, 84.5%). Consumers were less interested in comparing their personal health data with anonymous data from other patients (146/396, 36.9%) and with anonymous data from the Flemish population (146/394, 37.1%).

### Determinant Analysis of the Importance People Attribute to Obtaining Health Information When Using a Patient Portal

Bivariate linear regression showed that the importance people attributed to obtaining health information when using a patient portal to access health data (dependent) was significantly associated (cutoff *P*≤.10) with age, employment status, self-rated health status, the interest in shared decision making, the importance of being able to question the decisions of physicians, the difficulty in finding relevant health information, the difficulty in assessing the reliability of health information, and the difficulty in understanding health information. Due to the collinearity between age and employment status, only age was used for multivariate analysis.

**Table 3:** Patient portal use characteristics (N=433).

Patient portal characteristics		Participants, n (%)^a^
Interested in using a patient portal		369 (89.6)
**Types of information people would prefer to have in their patient portal**		
	My allergies	263 (64.1)
	Test results (eg, X-rays, blood tests, etc)	381 (92.9)
	Immunization records (list of vaccines received)	338 (82.4)
	Medication I have taken or am currently taking	345 (84.1)
	List of doctors or health care providers I have seen	279 (68.0)
	Family history of health problems	236 (57.6)
	Medical problems	322 (78.5)
	Medical visits or surgeries or medical procedures I have had	338 (82.4)
	Lifestyle choices (eg, smoking history and exercise)	138 (33.7)
	Information from devices that help me monitor my health	199 (48.5)
**Activities I am doing or** **would like to do on the internet**		
	View medical records, test results, and medication list	384 (94.8)
	Add notes to my medical record	312 (78.6)
	Request appointments, referrals, prescription refills	376 (93.3)
	Communicate with my doctor and receive reports by mail	358 (88.8)
	Fill out paperwork before or after a physician visit	331 (83.0)
	Sign up for reminders for preventive medicine (eg, flu shot)	360 (90.2)
	Learn about opportunities to participate in medical research	327 (82.6)
	Access my child’s or parents’ medical record if I am primary caretaker	315 (81.2)
	Communicate with other people with similar health problems	197 (50.8)
	Receive educational materials related to my health	304 (79.4)
	Record my representative to manage my health care when I am not able	337 (83.6)
**Expected frequency of patient portal use**		
	At least 1 time per week	61 (14.8)
	1 time per month	159 (38.6)
	Every 3-6 months	159 (38.6)
	Rarely or never	33 (8.0)
**How many people think the use of a patient portal will improve the following^b^**		
	Security and privacy of my medical information	90 (22.3)
	Communication between my doctors and myself	321 (78.7)
	My understanding of my own health	300 (73.7)
	My sense of control over my own health care	311 (76.4)
	My worries about my own health care	236 (58.1)
	The safety of my care (freedom from errors)	278 (68.6)
	My satisfaction with my health care	275 (68.1)
	The overall quality of my health care	292 (72.1)
	The overall costs of my health care	192 (47.4)
**Difficulty to use a patient portal to view and manage your health information and care**		
	Difficult	22 (5.3)
	Easy	292 (70.9)
	Neutral	98 (23.8)
**Who would you give permission to view information in your patient portal**		
	Designated family members or friends	226 (54.6)
	My primary care doctor	391 (94.4)
	Other doctors or health care providers who care for me	332 (80.2)
	My health insurance	26 (6.3)
	My employer	1 (0.2)
	The government	8 (1.9)
	No-one	18 (4.3)
**Importance of certain features when using a patient portal^c^**		
	Compare recent personal health data with health data from the past	311 (78.1)
	Compare personal health data with medical standards	291 (73.5)
	Compare personal health data with anonymous data from other patients	146 (36.9)
	Compare personal health data with anonymous data from the Flemish population	146 (37.1)
	See connections between your symptoms, your disease(s), your biological parameters	339 (85.2)
	See connections between your health and the presence of environmental factors	284 (71.7)
	View the evolution of your health in function of time	333 (84.5)
	View information about the expected effect of treatment on your personal health	349 (88.4)
	View information on the expected impact of your lifestyle on your personal health	309 (79.0)
	Provide your data anonymously so that regional problems can be detected	250 (63.1)
	Be notified when certain physical parameters evolve toward dangerous levels	370 (93.7)
	Be notified when your health changes	371 (93.7)

^a^Numbers may not sum to totals due to missing data. Percentages were calculated considering the missing data.

^b^Used as a composite dependent variable: “expectation concerning one’s personal health care when using a patient portal.”

^c^Used as a composite dependent variable: “importance attached to obtaining health information when using a patient portal to access health data.”

In the multivariate linear regression, age, shared decision making, and the importance of being able to question the decisions of physicians were significant (cutoff *P*≤.05). People attributed greater importance to receiving health information if they were aged 45-54 years compared with those who were aged 18-34 years, if they had an interested or neutral attitude toward shared decision making compared with having a negative attitude toward shared decision making and if they found it important to be able to question the decisions made by physicians compared with finding this unimportant (*R*^2^=0.122; Table 4).

### Determinant Analysis of Expectations Concerning Personal Health Care When Using a Patient Portal

Bivariate linear regression showed that expectations concerning personal health care when using a patient portal (dependent) were significantly associated (cutoff *P*≤.10) with age, employment status, education, the interest in shared decision making, the importance of being able to question the decisions of physicians, the difficulty in finding relevant health information, the difficulty in assessing the reliability of health information, and the difficulty in understanding health information.

Due to the collinearity between age and employment status, only the employment status was used for multivariate analysis. Multivariate linear regression showed that expectations concerning personal health care when using a patient portal (dependent) was significantly associated (cutoff *P*≤.05) with education, shared decision making, the difficulty in finding relevant health information and problems in understanding health information (Table 4). People expected an improvement in their individual health care when using a patient portal if they had lower levels of education (high school degree or lower) compared with highly educated participants (master degree or higher), an interested or neutral attitude toward shared decision making compared with a negative attitude toward shared decision making and if they sometimes had problems to understand health information compared with rarely having problems in understanding health information. People expected an impairment in their health care when using a patient portal if they found it easy or had a neutral attitude toward finding relevant health information compared with thinking it is difficult to find this information (*R*^2^=0.106; Table 4).

### Determinant Analysis of the Interest in Using a Patient Portal

Owing to the low variance between the independent and dependent variables, the determinant analysis showed results with very high uncertainty and was not conclusive.

**Table 4:** Multivariate regression models.

Multivariate regression models			*P* value	Beta (95% CI)
**Importance of obtaining health information**				
	**Shared decision making**			
		Important	.03	–.558 (–0.969 to –0.147)
		Neutral	.008	–.500 (–0.952 to –0.048)
		Unimportant (constant)	—^a^	—
	**Questioning decisions of physicians**			
		Important	.03	–.642 (–1.222 to –0.061)
		Neutral	.12	–.467 (–1.052 to 0.119)
		Unimportant (constant)	—	—
	**Age^b^in years**			
		18-34 (constant)	—	—
		35-44	.069	–.186 (–0.386 to 0.014)
		45-54	.047	–.183 (–0.363 to –0.002)
		55-64	.10	–.126 (–0.278 to 0.026)
		>65	.099	–.124 (–0.271 to 0.023)
**Expectations concerning personal health care**				
	**Education**			
		High school or lower	.04	–.155 (–0.303 to –0.007)
		Bachelor degree	.84	–.015 (–0.153 to 0.124)
		Master degree or higher (constant)	—	—
	**Shared decision making**			
		Important	.008	–.566 (–0.983 to –0.148)
		Neutral	.03	–.521 (–0.989 to –0.052)
		Unimportant (constant)	—	—
	**Finding relevant health information**			
		Difficult (constant)	—	—
		Not difficult or not easy	.017	.180 (0.032 to 0.328)
		Easy	.022	.197 (0.028-0.366)
	**Problems in understanding health information**			
		Frequently	.99	.002 (–0.234 to 0.237)
		Sometimes	.037	–.141 (–0.272 to –0.009)
		Rarely (constant)	—	—

^a^Not applicable.

^b^Owing to collinearity with employment status, only age was used for the multivariate regression.

## Discussion

### Principal Findings

This study used a cross-sectional survey design to investigate health information needs, expectations, and interest of people accessing a patient portal to view their health data. The health information needs in this context are mainly features such as being notified when one’s health changes and being notified when physical parameters increase to dangerous levels. In addition, people are interested to see connections among their symptoms, disease, and biological parameters. Moreover, they prefer to have an overview of how their health evolves and to have access to information about the expected effect of treatment on their health. These findings are in line with the patients’ desire for direct communication through a patient portal that was described in previous work [32].

The importance that people attribute to obtaining health information when using a patient portal is partially predicted by age and the level of patient empowerment, namely the importance of shared decision making and questioning physicians’ decisions. Expectations concerning the impact on personal health care when using a patient portal are influenced by the level of education, interest in shared decision making, and the difficulty people experience in finding and understanding the relevant health care information. These findings confirm that screening for eHealth literacy and providing training in the use of a patient portal could help in improving the experience and expectations people have when using a patient portal [33,34].

People expect that the use of a patient portal can improve the communication between their physician and themselves. However, only a few people think that the use of a patient portal will improve the security and privacy of their medical information. In this study, the expressed interest in using a patient portal was high, with almost 90% of respondents interested in the use of a patient portal, although there was no functional patient portal available in Belgium at the time the survey was submitted. The fact that people in Flanders express the need to be notified when their health changes highlights the interest of people in receiving some form of communication or alert through their patient portal as to when they need to act to manage their health care. This corresponds with previous research that states that receiving intelligent alerts is an important feature in the conceptual design of an integrated shared decision personal health record [35]. A patient-directed clinical decision system that is integrated into a patient portal could be useful for this purpose as the ongoing research hypothesizes [36].

Although earlier research shows that incorporating patients’ lifestyle is important for patient portals to become more user-centric [37], only a third of our respondents were interested to have data about lifestyle choices in their patient portal. A potential explanation might be that people when conceptualizing a patient portal consider themselves to be aware of their lifestyle and therefore do not feel the need to find this information in a patient portal. Patients in Flanders, who have no experience with a patient portal, might think about a patient portal as a unidirectional channel where they can find and consult their health information, where people who have actually used a patient portal, value it as a bidirectional tool [11].

Another important finding is the fact that almost 9 of 10 respondents consider it important to be able to question the decisions made by physicians. This finding, together with the importance of shared decision making (93% consider this important), emphasizes the fact that people in Flanders are critical health care users who greatly appreciate patient empowerment and endorses the results from a former study where Belgian inhabitants attained fairly high empowerment scores [38].

Previous research showed that people think the use of a patient portal can improve the communication between their physician and themselves [39], the understanding of their own health [14], the sense of control over their health care [40], and the overall quality of their health care [4]. In literature, there is some skepticism about the influence of a patient portal on the security and privacy of health data and the total costs of health care [13,41,42]. People are mostly interested in test results, medication schemes, immunization records, and a history of medical visits and procedures [21,43]. These findings are consistent with ours, where about three-quarters of respondents believed that a patient portal could improve the doctor-patient communication, the understanding and sense of control of their health, and the quality of care.

Only a small percentage of respondents (22.3%) think the use of a patient portal can improve the privacy and security of medical information and almost half of them believe a patient portal can lower the cost of health care. People consider information about lifestyles choices and data from devices to help monitor their health to be the least interesting information in a patient portal. The lack of interest to have data in a patient portal that is gathered from devices to monitor one’s health corresponds with findings from a previous study, which states that “tools alone are not enough” and engaging patients in the use of a patient portal requires a patient-centered approach [44].

### Strengths and Limitations

This study is one of the first to investigate the health information needs of patients on conceptualizing a patient portal to access digital health data. In contrast to other studies in this field, which often focus on a functional patient portal, we had to start from the very beginning, as there was no functional patient portal available in Belgium at the time our survey was submitted [32,45-48]. In addition, the use of cultural probes and ideation techniques, frequently used in design and human computer interaction research, provides insights into the way people would like to interact with digital health data. It creates the opportunity to design a patient portal that considers the health information needs expressed by future users. Our survey was distributed across a large region in Belgium and included respondents of all age categories.

Despite our efforts to reduce selection bias to a minimum, we could not recruit participants from different ethnic backgrounds. Our respondents almost exclusively spoke Dutch. This is not in line with the demographics in Flanders, where a significant part of the population (8.1%) is of foreign origin. As almost 98% of respondents used the internet at least once a day, we could not find much evidence for the so-called “digital divide,” which is described in previous research as an important barrier in the implementation of a patient portal [49]. One potential explanation for this could be the Web-based nature of the survey and the fact that reminders to participate were mostly made with the help of social media and Web-based newsletters. This means our findings may not be representative of some groups in the Flemish population who rarely use the internet. Finally, our 2 statistical models had a low predictive power with an *R*^2^ of 0.122 (predicted importance people attribute to obtaining health information when using a patient portal) and.106 (predicted expectations toward personal health care when using a patient portal). Although these *R*^2^ values were low, this is not unusual in social research [50].

### Conclusions

This study yields a range of relevant aspects to consider when designing a patient portal. First of all, people express the need for a patient portal and personal access to it. Second, people like to receive alerts or some form of communication to foster their health management. Finally, several patient characteristics influence people’s attitude toward a patient portal. As such, middle-aged people and those with a positive attitude toward shared decision making attach more importance to obtaining health information. People with a lower education level or with low health literacy expect an improvement in their health care by using a patient portal.

## Appendix

Multimedia Appendix 1 Survey part 1 and part 2.

Multimedia Appendix 2 Survey part 1 and part 2, Dutch translation.

Multimedia Appendix 3 The Checklist for Reporting Results of Internet E-Surveys (CHERRIES).

Multimedia Appendix 4 Qualitative study that forms the basis for the second part of the survey.

Multimedia Appendix 5 Predictor variables for regression models.

## Abbreviations

HITEC: Health Information Technology Evaluation Collaborative
