# Supplementary material for: Designing a Patient Portal for Patient-Centered Care: Cross-Sectional Survey
Source: J Med Internet Res. 2018 Oct 1;20(10):e269. doi: 10.2196/jmir.9497 (PMC6231862; doi:10.2196/jmir.9497)
Supplement: Multimedia Appendix 1 [file jmir_v20i10e269_app1.pdf]

Multimedia Appendix 1: Survey part 1 and part 2.

Part 1 of the survey:

Questions 1-9: These questions ask about your health and use of healthcare services.

1) In general, how would you rate your overall health?

O	O	O	O	O
Excellent	Very good	Good	Fair	Poor

2) Are you being treated for any ongoing, persistent, or intermittent disease or medical condition such as high blood pressure, diabetes, heart or lung disease, mental health problem, or arthritis?
   O Yes
   O No

3) Are you taking any medication(s) prescribed by a doctor?
   O Yes
   O No

4) How many visits in the **past year** have you had with the following healthcare providers or facilities?

	None	1	2-3	More than 3
a) Primary Care Provider (including doctors and nurse practitioners)	O	O	O	O
b) Specialist (gynecologist, heart doctor, skin doctor, lung doctor, allergist, etc)	O	O	O	O
c) Emergency Department	O	O	O	O
d) Hospital (stayed at least overnight)	O	O	O	O

5) Overall, how satisfied are you with the quality of health care service you have received in the past **5 years**?

O	O	O	O	O
Very satisfied	Somewhat satisfied	Neutral	Somewhat dissatisfied	Very dissatisfied

6) How often do you have problems understanding your doctors when they are talking to you about your health?

O	O	O	O	O
Always	Often	Sometimes	Occasionally	Never

7) How often do you have problems understanding written medical information (such as forms, written instructions, or pamphlets) from your doctor or your doctor's office?

O	O	O	O	O
Always	Often	Sometimes	Occasionally	Never

8) Which of the following **best** describes the way you make decisions about your medical care? Please choose one:

   O My doctor(s) gives recommendations, but I make my own decisions about my medical care
   O My doctor(s) and I make decisions about my medical care together as a team
   O I leave it up to my doctor(s) to make the best decisions about my medical care

9) Are you currently caring for or making healthcare decisions for a family member or a close friend with a debilitating or chronic illness?
   O Yes
   O No

Questions 10-14: These questions ask about use of computers and the Internet.

10) Do you have a computer with Internet access available for use, either at home or work?
  O Yes
  O No

11) How often do you access the Internet?

O	O	O	O	O
Several times a day	About once a day	Once weekly	Once monthly	Rarely or not at all

12) What is your primary source of information for issues in your community?
  O Newspaper
  O Television
  O Radio
  O Internet

13) Which of the following activities have you done on the Internet? Please check all that apply:
  O Sent and received e-mails
  O Purchased something online with a credit card
  O Paid bills and/or managed bank accounts online
  O Looked up information on health and diseases

14) Do any of your doctors or healthcare providers currently use a computer to store and access your medical record?
  O Yes
  O No
  O Don't know

Questions 15-21: These questions ask about electronic **patient portals**. Unlike an electronic medical record that is primarily used by your healthcare providers, an electronic **patient portal is primarily used by you to view your health information and manage your healthcare (for example, to make appointments) on the Internet**.

15) Please indicate how strongly you agree or disagree with the following statement: I am interested in using a patient portal to view my health information and manage my healthcare.

O	O	O	O	O
Strongly agree	Agree	Neutral	Disagree	Strongly disagree

16) Which of the following types of health information would you like to have as part of your patient portal? Please check all that apply:
  O My allergies
  O Test results (examples: blood tests, X-rays)
  O Immunization records (a record of vaccines you have received)
  O Medications I have taken or am currently taking
  O List of doctors and healthcare providers I have seen
  O Family history of health problems
  O Medical problems
  O Medical visits, surgeries, and medical procedures that I have had
  O Lifestyle choices (example: exercise, smoking history)
  O Information from devices that help me monitor my health (example: glucose from a diabetes meter)

17) For each of the activities below, please indicate if this is an activity you already do, you don't currently do but would like to be able to do, or you do not want to do **on the Internet**:

	I do this on the Internet now	I would like to do this on the Internet	I would not do this on the Internet
a) View my medical records, test results, and lists of medications I am taking	O	O	O
b) Add notes to my medical record	O	O	O
c) Request medical appointments, referrals to other doctors, and prescription refills	O	O	O
d) Communicate with my doctor and/or receive reports from my doctor by e-mail	O	O	O
e) Find a physician who accepts my insurance, file insurance claims, and fill out paperwork before and after a physician visit	O	O	O
f) Sign up for reminders for preventative health services (such as the flu shot or cholesterol testing)	O	O	O
g) Learn about opportunities to participate in medical research	O	O	O
h) Access my child's or parent's medical records if I am their primary caretaker	O	O	O
i) Communicate with other people with similar health problems (examples: support groups, discussion forums, etc)	O	O	O
j) Receive educational materials related to my health	O	O	O
k) Record my selection of a family member or friend to manage my healthcare when I am not able to and record my treatment preferences (example: Do Not Resuscitate order)	O	O	O

18) How often do you think you would use a patient portal to view your health information and manage your health care (for example, take care of appointments and insurance issues and communicate with your doctors) on the Internet?

O	O	O	O	O
About once daily	Once weekly	Once monthly	Once every 3 to 6 months	Rarely or not at all

19) How do you think using a patient portal to view your health information and manage your health care on the Internet will affect the following:

	Greatly improve	Slightly improve	No effect	Slightly worsen	Greatly worsen
a) The security and privacy of my medical information	O	O	O	O	O
b) Communication between my doctors and myself	O	O	O	O	O
c) My understanding of my own health	O	O	O	O	O
d) My sense of control over my own healthcare	O	O	O	O	O
e) My worries about my own healthcare	O	O	O	O	O
f) The safety of my care (freedom from errors)	O	O	O	O	O
g) My satisfaction with my healthcare	O	O	O	O	O
h) The overall quality of my healthcare	O	O	O	O	O
i) The overall costs of my healthcare	O	O	O	O	O

20) How difficult or easy do you think it would be for you to use a patient portal to view your health information and manage your health care on the Internet?

O	O	O	O	O
Very difficult	Difficult	Somewhat difficult	Easy	Very easy

21) Who would you give permission to view information in your patient portal? Please check all that apply:

O Designated family members/friends
O My primary care doctor
O Other doctors or healthcare providers who care for me (in clinic, the ER, or the hospital) O My health plan
O My employer
O Government officials
O I would not give anyone permission

Questions 22-34: The following questions are to help us understand who is responding to this survey. This information will not be used to identify you individually.

22) How old are you? __________

23) Are you male or female?
O Male
O Female

24) Indicate which country you are born in? ____________

25) Indicate which country your mother was born in? _____________

26) Indicate which country your father was born in? ____________

27) What is the primary language spoken in your home?
O Dutch
O French
O German
O Other – Please state: ___________

28) What is the highest grade or level of school you have completed?
O Primary school
O High school
O Bachelor degree
O Master degree or higher

29) What is your employment status?
O Employed full or part time
O Self-employed
O Full time student
O Homemaker
O Disabled and not working
O Currently unemployed, not disabled
O Retired

30) Which describes the best estimate of your annual household income before taxes?
O Under 20,000 €
O 20,000-30,000 €
O 30,000-40,000 €
O 40,000-60,000 €
O 60,000-80,000 €
O More than 80,000 €
O I don‘t know
O I don’t wish to answer this question

31How many people, including yourself, live in your household? _______

32) How many children aged 18 or under live with you? _______

33) What is your current zip code? __ __ __ __

34) What is the best description of your current living situation
O Urban
O Rural

<u>Part 2 of the survey:</u>

Questions 1-5: These questions ask how you value matters related to health.

1) Indicate how important each of the following things is to you:

	Very important	Important	Not really important	Unimportant	Very unimportant
Being aware of all the details about your health					
Understand exactly what the cause is of the symptoms you experience					
Knowing whether you are healthy or not					

2) Indicate how important each of the following things is to you:

This question is only for participants who get treated for an acute, prolonged, or chronic condition such as high blood pressure, diabetes, heart and lung disease, mental problems, or arthritis.

	Very important	Important	Not really important	Unimportant	Very unimportant
Knowing exactly the nature of your condition					
Knowing exactly your prognosis					
Knowing exactly how your condition will evolve					
That people in your environment believe you are not imagining your symptoms					
Knowing the current and exact state of your condition					

3) How worried are you that you are not completely informed of all things related to your health?

O Very worried	O Worried	O Not very worried	O Not worried	O Not worried at all

4) How interesting is it to understand exactly how your body works exactly?

O Very interesting	O Interesting	O Not very interesting	O Uninteresting	O Very uninteresting

5) Indicate how strongly you agree or disagree with each of the following statements:

	Strongly agree	Agree	Neutral	Disagree	Strongly disagree
Sometimes, I wonder how common the symptoms are I experience.					
Sometimes, I wonder whether my symptoms are severe enough to take action					
Sometimes, I wonder whether there are symptoms I should watch out for, so I can take actions in time					

Questions 6-8: These questions ask about your health literacy.

6) Indicate how difficult or easy the following things is to you:

	Very difficult	Difficult	Somewhat difficult	Easy	Very easy
Find information that is relevant to your personal health situation					
Assess the reliability of the health information you find yourself					

7) How often do you have problems understanding the health information you find yourself?

O Always	O Often	O Sometimes	O Occasionally	O Never

8) Indicate how strongly you agree or disagree with each of the following statements:

	Strongly agree	Agree	Neutral	Disagree	Strongly disagree
The health information I can find is often too general and does not apply to my personal situation					
I have ever been disappointed in the results of tests or treatments					

Questions 9-11: These questions ask how you deal with your condition.

Questions 9–11 are only for participants who get treated for an acute, prolonged, or chronic condition such as high blood pressure, diabetes, heart and lung disease, mental problems, or arthritis.

9) Indicate how strongly you agree or disagree with each of the following statements:

	Strongly agree	Agree	Neutral	Disagree	Strongly disagree
I do everything possible to prevent me from crossing my borders					
Sometimes, I do something while I know in advance that I will need to recover from it for a few days					
Sometimes, I decide not to do something because I know in advance I will need to recover from it for a few days					
I do everything to prevent my symptoms to get worse					

10) How important is it for you to be able to form realistic expectations about the evolution of your condition?

O	O	O	O	O
Very important	Important	Not really important	Unimportant	Very unimportant

11) How difficult is it for you to deal with the emotional effects of your condition?

O Very difficult	O Difficult	O Somewhat difficult	O Easy	O Very easy

Questions 12-13: These questions ask how you value effective care.

12) Indicate how important each of the following things is to you:

	Very important	Important	Not really important	Unimportant	Very unimportant
Knowing exactly what the risks are before you undergoing a treatment					
Knowing exactly what the possible side effects are before you undergoing a treatment					
Knowing exactly what the expected effects are before you undergoing a treatment					
Knowing exactly what the chances of success are before you undergoing a treatment					
Knowing exactly which complications may occur before you undergoing a treatment					
Understanding exactly why the treatment is necessary before undergoing it					
Knowing exactly what the risks are before you undergoing a treatment					
Knowing in detail what will happen in your body before					

undergoing a treatment					
Understanding exactly what treatment is most appropriate for your personal situation					

13) Indicate how strongly you agree or disagree with each of the following statement: I do everything to undergo as few treatments as possible.

O	O	O	O	O
Strongly agree	Agree	Neutral	Disagree	Strongly disagree

Questions 14-15: These questions ask about your wishes regarding the relationship with your doctors.

14) Indicate how strongly you agree or disagree with each of the following statement: It is important to me that my doctor and I make decisions about my medical care together, and not the doctor on their own.

O	O	O	O	O
Strongly agree	Agree	Neutral	Disagree	Strongly disagree

15) How important is it for you, in your relationship with your doctor, that you can question their decisions?

O	O	O	O	O
Very important	Important	Not really important	Unimportant	Very unimportant

Questions 16-18: These questions ask how you value good health.

16) Indicate how strongly you agree or disagree with each of the following statement: I do everything to attain or good health or maintain it.

O	O	O	O	O
Strongly agree	Agree	Neutral	Disagree	Strongly disagree

17) How worried are you that there are factors that influence your health without realizing them?

O	O	O	O	O
Very worried	Worried	Not very worried	Not worried	Not worried at all

18) Indicate how important each of the following things is to you:

	Very important	Important	Not really important	Unimportant	Very unimportant
Understanding exactly what impact your diet has on your health					
Understanding exactly what impact your physical activity has on your health					
Understanding exactly what impact					

environmental factors have on your health					

Questions 19-20: These questions ask how you value patient rights.

19) Indicate how worried you are for each of the following statements:

	Very worried	Worried	Not very worried	Not worried	Not worried at all
That your rights as a patient are violated					
That you are treated differently than others					
About your future social and financial security					

20) Indicate how strongly you agree or disagree with each of the following statements:

	Strongly agree	Agree	Neutral	Disagree	Strongly disagree
I know exactly which reimbursements I am entitled to					
I know exactly which support I am entitled to					
My future social and financial security are clear to me					

Questions 21-22: These questions ask to what extend you feel recognized regarding your health.

21) Indicate how strongly you agree or disagree with each of the following statements:

	Strongly agree	Agree	Neutral	Disagree	Strongly disagree
I do not always feel taken seriously by my doctors					
I do not always feel taken seriously regarding my health by people around me					

22) How important is it for you that people around you understand how it is to live with your condition?

O<br>Very important	O<br>Important	O<br>Not really important	O<br>Unimportant	O<br>Very unimportant

Questions 23-25: These questions ask how you value insight in your medical history.

23) Indicate how important each of the following things is to you:

	Very important	Important	Not really important	Unimportant	Very unimportant
Knowing exactly what complications occurred after a surgery, injury, or treatment					
Knowing exactly what the effects will be after a surgery, injury, or treatment					

24) How difficult is it for you to keep an overview on the appointments, treatments, and surgeries you had in the past?

O<br>Very difficult	O<br>Difficult	O<br>Somewhat difficult	O<br>Easy	O<br>Very easy

25) Indicate how strongly you agree or disagree with each of the following statement: Sometimes, I wonder whether test results are correct.

O<br>Strongly agree	O<br>Agree	O<br>Neutral	O<br>Disagree	O<br>Strongly disagree

Questions 26-29: These questions ask about your current experiences with healthcare.

26) Indicate how strongly you agree or disagree with each of the following statements:

	Strongly agree	Agree	Neutral	Disagree	Strongly disagree
Sometimes, I have the impression that my doctors do not tell me everything they know about my health					
My doctors do not always explain their decisions well enough					
Sometimes, I have the impression that my doctors do					

not look at the overall picture of my health, but reason too much from their own specialism					
It happens that my doctors are not well aware of my situation, so I have to repeat my story over and over again					
Before I visit my doctor, I try to find relevant information to be well prepared					
It happens that after my visit to my doctor, I notice that I did not ask every questions I had					
From time to time, I track some health information (e.g. a pain, sleep, or diet diary, my weight, sports, …)					

27) Indicate how important each of the following things is to you:

	Very important	Important	Not really important	Unimportant	Very unimportant
Being well prepared before visiting a doctor					
After a visit to your doctor, seek additional information or evidence about what your doctor has discussed with you					
Receive the information that your doctors give you in a tangible way (not only verbally)					

28) How often do you have problems remembering details of what your doctor discussed with you about your health?

O	O	O	O	O
Always	Often	Sometimes	Occasionally	Never

29) Indicate how difficult or easy the following things is to you:

	Very difficult	Difficult	Somewhat difficult	Easy	Very easy
After you have visited your doctor, seek additional information or evidence about what your doctor has discussed with you					
Manage administrative things about your health (e.g. appointments with doctors or other healthcare providers).					

Questions 30-34: These questions ask about your experiences with a patient portal.

30) Indicate how strongly you agree or disagree with each of the following statements:

	Strongly agree	Agree	Neutral	Disagree	Strongly disagree
I have ever wondered what information was in my patient portal					
I have ever had the impression that the content of my patient portal is not correct					
I have ever had the impression that there was information in my patient portal of which I was not aware of					
It is clear to me who has access to the data in my patient portal					
It is clear to me what happens					

with the data in my patient portal					
If I were asked, I would agree that the (anonymized) data in my patient portal could be used for research by scientific institutions such as universities					
If I were asked, I would agree that the (anonymized) data in my patient portal could be used for research by commercial institutions (e.g. pharmaceutical companies, insurers, etc.)					

31) Indicate how worried you are for each of the following statements:

	Very worried	Worried	Not very worried	Not worried	Not worried at all
That there are people who have access to the data in my patient portal, whom I prefer they would not have access to it					
That the data in my patient portal can be used and distributed without permission					
That the data in my patient portal can be used for commercial benefit					
That my insurance / mutuality would treat me differently if they had access to the data in my patient portal					
That my insurance /					

mutuality would exclude me from insurance if they would have access to the data in my patient portal					

32) Have you ever asked for access to your patient portal through a doctor or other healthcare provider?

O<br>Yes	O<br>No	O<br>I don’t know

Question 33 is only for participants who answered ‘Yes’ to question 32.

33) Indicate how strongly you agree or disagree with each of the following statement: It was easy to get access to my patient portal.

O<br>Strongly agree	O<br>Agree	O<br>Neutral	O<br>Disagree	O<br>Strongly disagree

34) How important is it for you that you can decide who gets access to the data in your patient portal?

O<br>Very important	O<br>Important	O<br>Not really important	O<br>Unimportant	O<br>Very unimportant

Questions 35-37: These questions ask about your wishes in case you could easily access all the data in your patient portal

35) Indicate how important each of the following things is to you, if you could have access to all the data in your patient portal:

	Very important	Important	Not really important	Unimportant	Very unimportant
Compare recent personal data with personal data from the past					
Compare your personal data with (medical) standards					
Compare your data with the (anonymized) data of other patients					
Compare your data with the (anonymized) data of the Flemish population					
Check for relationships between your symptoms, your condition(s), your biological parameters, etc.					
Check for relationships					

between your health and the presence of certain environmental factors					
Check the evolution of your health over time					
Check information about the expected effect of treatments on your health					
Check information about the expected impact of your lifestyle on your health					
Make your (anonymized) data available so that regional or rural issues can be detected					
Being notified when certain physical parameters evolve towards dangerous levels					
Being notified when your health situation changes					

36) If you had access to all the data in your patient portal, how difficult or easy do you think it would be to compare your data to the (anonymized) data from others?

O	O	O	O	O
Very difficult	Difficult	Somewhat difficult	Easy	Very easy

37) Indicate how strongly you agree or disagree with each of the following statements:

	Strongly agree	Agree	Neutral	Disagree	Strongly disagree
If I could compare the data in my patient portal with the (anonymized) data from others on my own, I would probably make mistakes that lead to false conclusions					
If I would have access to all data in my patient portal, I would probably have unnecessary worries					
