# Supplementary material for: Designing a Patient Portal for Patient-Centered Care: Cross-Sectional Survey
Source: J Med Internet Res. 2018 Oct 1;20(10):e269. doi: 10.2196/jmir.9497 (PMC6231862; doi:10.2196/jmir.9497)
Supplement: Multimedia Appendix 2 [file jmir_v20i10e269_app2.pdf]

Multimedia Appendix 2: Survey part 1 and part 2

Dutch translation

Part 1

Vraag 1-9: Deze vragen informeren naar uw gezondheid en uw gebruik van de gezondheidszorg.

1) Hoe zou u, over het algemeen, uw gezondheid beschrijven?
   - Uitstekend
   - Zeer goed
   - Goed
   - Redelijk
   - Slecht

2) Wordt u behandeld voor een acute, langdurige, of chronische aandoening zoals hoge bloeddruk, diabetes, hart- en longziekten, mentale problemen, of artritis?
   - Ja
   - Nee

3) Gebruikt u medicatie voorgeschreven door een arts?
   - Ja
   - Nee

4) Hoe vaak heeft u in het **afgelopen jaar** de volgende zorgverleners of zorgverlenende instanties bezocht?

	nooit	1 keer	2-3 keer	meer dan 3 keer
Eerstelijns zorgverleners (huisarts, kinesist, diëtist, etc.)				
Specialisten (gynaecoloog, cardioloog, dermatoloog, longarts, etc.)				
Spoeddienst				
Ziekenhuis (opname van tenminste één nacht)				

5) Hoe tevreden bent u, in het algemeen, met de kwaliteit van de gezondheidszorg die u de afgelopen **5 jaar** heeft gebruikt?

- Zeer tevreden
- Enigszins tevreden
- Neutraal
- Enigszins ontevreden
- Zeer ontevreden

6) Hoe vaak heeft u problemen om uw arts te begrijpen wanneer hij/zij met u spreekt over uw gezondheid?
   - Altijd
   - Vaak
   - Soms
   - Zelden
   - Nooit

7) Hoe vaak heeft u problemen om geschreven medische informatie (zoals formulieren, geschreven instructies, of folders) van uw arts te begrijpen?
   - Altijd
   - Vaak
   - Soms
   - Zelden
   - Nooit

8) Welke van onderstaande uitspraken beschrijft het best hoe u beslissingen neemt over uw medische zorg? <u>Gelieve er één te kiezen:</u>
   - Mijn arts geeft me aanbevelingen, maar ik neem mijn eigen beslissingen over mijn medische zorg
   - Mijn arts en ik nemen samen beslissingen over mijn medische zorg, als een team
   - Ik laat het aan mijn arts over om de beste beslissing te nemen over mijn medische zorg

9) Zorgt u momenteel voor, of neemt u beslissingen over de gezondheidszorg van een familielid of goede vriend die lijdt aan een ernstige of chronische ziekte?
   - Ja
   - Nee

Vraag 10 – 14: Deze vragen gaan over het gebruik van computers en het internet.

10) Heeft u, thuis en/of op het werk, een computer met internetverbinding tot uw beschikking?
    - Ja
    - Nee

11) Hoe vaak gebruikt u het internet?
    - Meerdere keren per dag
    - Ongeveer 1 keer per dag
    - Een keer per week

- Een keer per maand
- Zelden of nooit

12) Wat is voor u de belangrijkste bron voor informatie uit uw omgeving?
    - Krant
    - Televisie
    - Radio
    - Internet

13) Welke van de volgende activiteiten heeft u al eens gedaan via het internet? <u>Meerdere opties mogelijk:</u>
    - Versturen en ontvangen van e-mails
    - Iets online aankopen en betalen met een credit card
    - Rekeningen betalen of online bankrekeningen beheren
    - Informatie opzoeken over gezondheid en ziekte

14) Gebruiken uw artsen of zorgverleners momenteel een computer om uw medische gegevens op te slaan en te gebruiken?
    - Ja
    - Nee
    - Weet ik niet

Vraag 15-21: Deze vragen gaan over elektronische **persoonlijke patiëntendossiers**. In tegenstelling tot een elektronisch patiëntendossier dat voornamelijk door uw zorgverleners wordt gebruikt, wordt een **elektronisch persoonlijk patiëntendossier vooral door uzelf gebruikt om via het internet informatie over uw gezondheid te bekijken en uw gezondheidszorg te beheren (bijvoorbeeld om afspraken te maken)**.

15) Duid aan in hoeverre u akkoord of niet akkoord gaat met de volgende stelling: Ik ben geïnteresseerd in het gebruik van een persoonlijk patiëntendossier om informatie over mijn gezondheid in te zien en mijn gezondheidszorg te beheren via het internet.
    - Helemaal akkoord
    - Akkoord
    - Neutraal
    - Niet akkoord
    - Helemaal niet akkoord

16) Welke informatie over uw gezondheid zou u willen terugvinden in uw persoonlijk patiëntendossier? <u>Meerdere opties mogelijk:</u>
    - Mijn allergieën
    - Testresultaten (voorbeelden: bloedtests, röntgenfoto's)
    - Mijn vaccinaties
    - Medicatie die ik heb gebruikt of momenteel gebruik
    - Lijst van artsen en zorgverleners die ik heb bezocht
    - Familiale geschiedenis van gezondheidsproblemen
    - Medische problemen

- Consultaties, operaties en medische procedures die ik heb ondergaan
- Lifestyle-keuzes (voorbeeld: beweging, roken)
- Informatie van toestellen die mijn gezondheid meten (voorbeeld: suikerwaarde via een glucosemeter)

17) Duid voor elk van onderstaande activiteiten aan of dit een activiteit is die u al doet, die u momenteel niet doen maar wel zou willen kunnen doen, of die u niet wilt doen **via het internet**:

	Ik doe dit nu al via het internet	Ik doe dit nu al via het internet	Ik doe dit nu al via het internet
Inzien van mijn medisch dossier, testresultaten en medicatielijst			
Notities toevoegen aan mijn medisch dossier			
Medische afspraken, doorverwijzingen naar andere artsen en vernieuwing van voorschriften opvragen			
Communicatie met mijn arts en/of verslagen van mijn arts via e-mail ontvangen			
Papierwerk invullen voor en na een doktersbezoek			
Aanmelden voor herinneringen aan preventieve gezondheidszorg (zoals de grieppprik)			
Geïnformeerd worden over mogelijkheden om deel te nemen aan medisch onderzoek			
Toegang hebben tot medische dossiers van mijn kinderen of ouders als ik hun voornaamste mantelzorger ben			
Communiceren met mensen met gelijkaardige gezondheidsproblemen (voorbeelden: zelfhulpgroepen, discussiefora, etc.)			
Ontvangen van voorlichtingsmateriaal gerelateerd aan mijn gezondheid			
Vastleggen wie van mijn familieleden of vrienden als vertegenwoordiger mag optreden als ik niet in staat ben om mijn wensen over behandelingen kenbaar te maken			

18) Hoe vaak denkt u dat u een persoonlijk patiëntendossier zou gebruiken om informatie over uw gezondheid in te zien en uw gezondheidszorg te beheren (bijvoorbeeld het maken van afspraken en communicatie met uw artsen) via het internet?
    - Ongeveer een keer per dag
    - Een keer per week
    - Een keer per maand
    - Elke 3 tot 6 maanden
    - Zelden of nooit

19) Hoe denkt u dat het gebruiken van een persoonlijk patiëntendossier om informatie over uw gezondheid in te zien en uw gezondheidszorg te beheren via het internet de volgende zaken zal beïnvloeden?

	Sterke verbetering	Sterke verbetering	Sterke verbetering	Sterke verbetering	Sterke verbetering
De beveiliging en privacy van mijn medische gegevens					
Communicatie tussen mijn artsen en mijzelf					
Het begrijpen van mijn eigen gezondheid					
Mijn gevoel van controle over mijn eigen gezondheid					
Mijn zorgen over mijn eigen gezondheid					
De veiligheid van mijn zorg (het uitblijven van fouten)					
Mijn tevredenheid over mijn gezondheidszorg					
De kwaliteit van mijn gezondheidszorg in het algemeen					

De kosten van mijn gezondheidszorg in het algemeen					

20) Hoe moeilijk of makkelijk denkt u dat het zou zijn om een persoonlijk patiëntendossier te gebruiken om informatie over uw gezondheid in te zien en uw gezondheidszorg te beheren via het internet?
    - Zeer moeilijk
    - Moeilijk
    - Enigszins moeilijk
    - Makkelijk
    - Zeer makkelijk

21) Wie zou u toestemming geven om informatie uit uw persoonlijke patiëntendossier te bekijken? <u>Meerdere opties mogelijk:</u>
    - Aangewezen familieleden of vrienden
    - Mijn huisarts
    - Andere artsen of zorgverleners die voor mij zorgen (bv. op de spoeddienst of in het ziekenhuis)
    - Mijn ziekteverzekeraar
    - Mijn werkgever
    - Overheid
    - Ik zou niemand toestemming geven

Vraag 22-34: De volgende vragen helpen ons te begrijpen wie deze vragenlijst invult. Deze informatie wordt niet gebruikt om u individueel te identificeren.

22) Hoe oud bent u? (open veld)

23) Bent u een man of een vrouw?
    - Man
    - Vrouw

24) Duid aan in welk land u geboren bent (open veld)

25) Duid aan in welk land uw moeder geboren is (open veld)

26) Duid aan in welk land uw vader geboren is (open veld)

27) Wat is de taal die meestal wordt gesproken bij u thuis?
    - Nederlands
    - Frans
    - Duits
    - Anders, namelijk

28) Wat is uw hoogst behaalde diploma?
   - Lagere school
   - Middelbare school
   - Bachelor-diploma
   - Master-diploma of hoger

29) Wat is uw werksituatie:
   - Voltijds of deeltijds in loondienst
   - Zelfstandige
   - Voltijds student
   - Huisvrouw of –man
   - Invalide
   - Momenteel werkloos, niet invalide
   - Gepensioneerd

30) Wat is dit jaar uw bruto gezinsinkomen?
   - Minder dan €20.000
   - €20.000 - €30.000
   - €30.000 - €40.000
   - €40.000 - €60.000
   - €60.000 – €80.000
   - meer dan €80.000
   - Weet ik niet
   - Ik wens deze vraag niet te beantwoorden

31) Uit hoeveel mensen, inclusief uzelf, bestaat uw huishouden? (open veld)

32) Hoeveel kinderen jonger dan 18 jaar wonen bij u? (open veld)

33) Wat is uw huidige postcode? (open veld)

34) Wat is de beste beschrijving van uw huidige woonsituatie:
   - Verstedelijkt gebied
   - Landelijk gebied

Part 2

Vraag 1-5 : Deze vragen informeren naar het belang dat u hecht aan zaken gerelateerd aan gezondheid.

1. Duid aan hoe belangrijk de volgende zaken voor u zijn:

	Zeer belang-rijk	Belang-rijk	Niet erg belang-rijk	Onbe-langrijk	Zeer onbe-langrijk
Op de hoogte zijn van alle details over uw gezondheid					
Precies begrijpen wat de oorzaak is van symptomen die u heeft					
Weten of u gezond bent of niet					

2. Duid aan hoe belangrijk de volgende zaken voor u zijn:

Vraag 2 is alleen zichtbaar voor deelnemers die 'ja' hebben geantwoord op vraag 2 uit deel 1 (Wordt u behandeld voor een acute, langdurige, of chronische aandoening zoals hoge bloeddruk, diabetes, hart- en longziekten, mentale problemen, of artritis?)

	Zeer belang-rijk	Belang-rijk	Niet erg belang-rijk	Onbe-langrijk	Zeer onbe-langrijk
Weten wat de aard van uw aandoening precies is					
Weten wat uw prognose precies is voor de toekomst					
Precies weten hoe uw conditie zal evolueren					
Dat mensen in uw omgeving geloven dat u zich uw symptomen niet inbeeldt					
Weten hoe het momenteel precies met uw aandoening is gesteld					

3. Hoe bezorgd bent u dat u niet volledig op de hoogte bent van alle zaken die ertoe doen m.b.t. uw gezondheid?
   - o Zeer bezorgd
   - o Bezorgd
   - o Niet erg bezorgd
   - o Niet bezorgd
   - o Totaal niet bezorgd

4. Hoe interessant vindt u het om begrijpen hoe uw lichaam precies werkt?
    - Zeer interessant
    - Interessant
    - Niet erg interessant
    - Oninteressant
    - Zeer oninteressant

5. Duid aan in hoeverre u akkoord of niet akkoord gaat met de volgende stellingen:

	Hele-maal akkoord	Akkoord	Neutraal	Niet akkoord	Hele-maal niet akkoord
Ik vraag me weleens af hoe gebruikelijk de symptomen zijn die ik heb.					
Ik vraag me weleens af of symptomen die ik heb ernstig genoeg zijn om actie te ondernemen of niet					
Ik vraag me weleens af of er symptomen zijn waar ik op zou moeten letten zodat ik op tijd actie kan ondernemen					

Vraag 6-8 : Deze vragen informeren uw gezondheidskennis.

6. Duid aan hoe moeilijk of makkelijk de volgende zaken voor u zijn:

	Zeer moeilijk	Moeilijk	Niet moeilijk maar ook niet makke-lijk	Makke-lijk	Zeer makke-lijk
Informatie vinden die relevant is voor uw persoonlijke gezondheidssituatie					
Inschatten hoe betrouwbaar de gezondheidsinformatie is die u zelf vindt					

7. Hoe vaak heeft u problemen om de gezondheidsinformatie die u zelf vindt te begrijpen?
    - Altijd
    - Vaak
    - Soms
    - Zelden
    - Nooit

8. Duid aan in hoeverre u akkoord of niet akkoord gaat met de volgende stellingen:

	Hele-maal akkoord	Akkoord	Neutraal	Niet akkoord	Hele-maal niet akkoord
De gezondheidsinformatie die ik kan vinden is vaak te algemeen en niet van toepassing op mijn persoonlijke situatie					
Ik ben weleens teleurgesteld geweest in de resultaten van tests of behandelingen					

Vraag 9-11: Deze vragen informeren naar hoe u omgaat met uw aandoening.

Vragen 9 t/m 11 zijn alleen zichtbaar voor deelnemers die 'ja' hebben geantwoord op vraag 2 uit deel 1 (Wordt u behandeld voor een acute, langdurige, of chronische aandoening zoals hoge bloeddruk, diabetes, hart- en longziekten, mentale problemen, of artritis?)

9. Duid aan in hoeverre u akkoord of niet akkoord gaat met de volgende stellingen:

	Hele-maal akkoord	Akkoord	Neutraal	Niet akkoord	Hele-maal niet akkoord
Ik doe er alles aan om te voorkomen dat ik over mijn grenzen ga					
Ik doe wel eens iets waarvan ik bij voorbaat weet dat ik er een paar dagen van zal moeten herstellen					
Ik beslis wel eens om iets niet te doen omdat ik bij voorbaat weet dat ik er een paar dagen van zal moeten herstellen					
Ik doe er alles aan om te voorkomen dat mijn symptomen verergeren					

10. Hoe belangrijk is het voor u om realistische verwachtingen te kunnen vormen over de evolutie van uw aandoening?
    - o Zeer belangrijk
    - o Belangrijk
    - o Niet erg belangrijk
    - o Onbelangrijk
    - o Zeer onbelangrijk

11. Hoe moeilijk is het voor u om met de emotionele gevolgen van uw aandoening om te gaan?
    - o Zeer moeilijk
    - o Moeilijk
    - o Niet moeilijk maar ook niet makkelijk
    - o Makkelijk
    - o Zeer makkelijk

Vraag 12- 13 : Deze vragen informeren naar het belang dat u hecht aan effectieve zorg.

12. Duid aan hoe belangrijk de volgende zaken voor u zijn:

	Zeer belang-rijk	Belang-rijk	Niet erg belang-rijk	Onbe-langrijk	Zeer onbe-langrijk
Precies weten wat de risico's zijn voordat u een behandeling ondergaat					
Precies weten wat de mogelijke bijwerkingen zijn voordat u een behandeling ondergaat					
Precies weten wat de verwachte effecten zijn voordat u een behandeling ondergaat					
Precies weten hoe groot de kans van slagen is voordat u een behandeling ondergaat					
Precies weten welke complicaties mogelijk kunnen optreden voordat u een behandeling ondergaat					
Precies begrijpen waarom een behandeling noodzakelijk is voordat u deze ondergaat					
Tot in detail weten wat er allemaal in uw lichaam gaat gebeuren voordat u een behandeling ondergaat					
Precies begrijpen welke behandeling voor uw persoonlijke situatie de meest geschikte is					

13. Duid aan in hoeverre u akkoord of niet akkoord gaat met de volgende stelling: Ik doe er alles aan om te zo weinig mogelijk behandelingen te ondergaan.
    - Helemaal akkoord
    - Akkoord
    - Neutraal
    - Niet akkoord
    - Helemaal niet akkoord

Vraag 14- 15 : Deze vragen informeren naar uw wensen m.b.t. de relatie met uw artsen.

14. Duid aan in hoeverre u akkoord of niet akkoord gaat met de volgende stelling: Het is belangrijk voor mij dat mijn arts en ik samen, en niet mijn arts alleen, beslissingen nemen over mijn medische zorg.
    - Helemaal akkoord
    - Akkoord
    - Neutraal
    - Niet akkoord
    - Helemaal niet akkoord

15. Hoe belangrijk is het voor u, in uw relatie met uw arts, dat u zijn/haar beslissingen ter discussie durft te stellen?
    - Zeer belangrijk
    - Belangrijk

- Niet erg belangrijk
- Onbelangrijk
- Zeer onbelangrijk

Vraag 16-18 : Deze vragen informeren naar het belang dat u hecht aan een goede gezondheid.

16. Duid aan in hoeverre u akkoord of niet akkoord gaat met de volgende stelling: Ik doe er alles aan om een zo goed mogelijke gezondheid te bereiken en/of te behouden.
    - Helemaal akkoord
    - Akkoord
    - Neutraal
    - Niet akkoord
    - Helemaal niet akkoord

17. Hoe bezorgd bent u dat er factoren zijn die uw gezondheid beïnvloeden zonder dat u zich dat beseft?
    - Zeer bezorgd
    - Bezorgd
    - Niet erg bezorgd
    - Niet bezorgd
    - Totaal niet bezorgd

18. Duid aan hoe belangrijk de volgende zaken voor u zijn:

	Zeer belang-rijk	Belang-rijk	Niet erg belang-rijk	Onbe-langrijk	Zeer onbe-langrijk
Precies begrijpen welke impact uw voedingspatroon heeft op uw gezondheid					
Precies begrijpen welke impact uw bewegingspatroon heeft op uw gezondheid					
Precies begrijpen welke impact omgevingsfactoren hebben op uw gezondheid					

Vraag 19-20: Deze vragen informeren naar het belang dat u hecht aan patiëntenrechten.

19. Duid aan hoe bezorgd u bent over de volgende zaken:

	Zeer bezorgd	Bezorgd	Niet erg bezorgd	Niet bezorgd	Hele-maal niet bezorgd
Dat uw rechten als patiënt geschonden worden					
Dat u als patiënt anders wordt behandeld dan anderen					

Uw sociale en financiële zekerheid in de toekomst					

20. Duid aan in hoeverre u akkoord of niet akkoord gaat met de volgende stellingen:

	Hele-maal akkoord	Akkoord	Neutraal	Niet akkoord	Hele-maal niet akkoord
Ik weet precies op welke terugbetalingen ik recht heb					
Ik weet precies op welke vormen van ondersteuning ik recht heb					
Het is mij duidelijk hoe het staat met mijn sociale en financiële zekerheid in de toekomst					

Vraag 21-22: Deze vragen informeren naar de mate waarin u zich erkend voelt als het gaat over uw gezondheid.

21. Duid aan in hoeverre u akkoord of niet akkoord gaat met de volgende stellingen:

	Hele-maal akkoord	Akkoord	Neutraal	Niet akkoord	Hele-maal niet akkoord
Ik voel me niet altijd serieus genomen door mijn artsen.					
Ik voel me niet altijd serieus genomen door mensen in mijn omgeving als het gaat over mijn gezondheid.					

22. Hoe belangrijk is het voor u dat mensen in uw omgeving begrijpen hoe het is om met uw aandoening te leven?
    - Zeer belangrijk
    - Belangrijk
    - Niet erg belangrijk
    - Onbelangrijk
    - Zeer onbelangrijk

Vraag 23-25: Deze vragen informeren naar de mate waarin u belang hecht aan inzicht in uw medische historiek

23. Duid aan hoe belangrijk de volgende zaken voor u zijn:

	Zeer belang-rijk	Belang-rijk	Niet erg belang-rijk	Onbe-langrijk	Zeer onbe-langrijk
Na afloop van een ingreep, ongeval of behandeling precies weten welke complicaties zijn opgetreden					
Na afloop van een ingreep, ongeval of behandeling precies weten wat de gevolgen zullen zijn					

24. Hoe moeilijk is het voor u om het overzicht te houden van afspraken, behandelingen en ingrepen die u in het verleden heeft gehad?
    - Zeer moeilijk
    - Moeilijk
    - Niet moeilijk maar ook niet makkelijk
    - Makkelijk
    - Zeer makkelijk

25. Duid aan in hoeverre u akkoord of niet akkoord gaat met de volgende stelling: Ik vraag mij weleens af of mijn testresultaten wel kloppen.
    - Helemaal akkoord
    - Akkoord
    - Neutraal
    - Niet akkoord
    - Helemaal niet akkoord

Vraag 26-29: Deze vragen informeren naar uw huidige ervaringen met gezondheidzorg

26. Duid aan in hoeverre u akkoord of niet akkoord gaat met de volgende stellingen:

	Hele-maal akkoord	Akkoord	Neutraal	Niet akkoord	Hele-maal niet akkoord
Ik heb weleens de indruk dat mijn artsen me niet alles vertellen dat ze weten over mijn gezondheid					
Mijn artsen leggen me hun beslissingen niet altijd goed genoeg uit					
Ik heb weleens de indruk dat mijn artsen onvoldoende kijken naar het totaalplaatje van mijn gezondheid, maar te veel vanuit hun eigen specialisme redeneren<br>Het gebeurt weleens dat mijn artsen niet goed op de hoogte zijn van mijn situatie waardoor ik steeds opnieuw mijn verhaal moet doen					
Voordat ik mijn arts bezoek probeer ik relevante informatie op te zoeken om goed voorbereid te zijn					

Het gebeurt wel eens dat ik na afloop van een bezoek aan mijn arts merk dat ik niet alle vragen heb gesteld die ik had					
Ik houd zelf weleens informatie bij over mijn gezondheid (bv. een pijn-, slaap- of eetdagboek, mijn gewicht, hoe veel ik beweeg, ...)					

27. Duid aan hoe belangrijk de volgende zaken voor u zijn:

	Zeer belang-rijk	Belang-rijk	Niet erg belang-rijk	Onbe-langrijk	Zeer onbe-langrijk
Goed voorbereid zijn voordat u uw arts bezoekt					
Na afloop van een bezoek aan uw arts extra informatie of duiding op kunnen zoeken over datgene dat uw arts met u heeft besproken					
De informatie die uw artsen u geven op een tastbare manier (dus niet alleen mondeling) ontvangen					

28. Hoe vaak heeft u problemen om de details te onthouden van wat uw arts met u bespreekt over uw gezondheid?
    - Altijd
    - Vaak
    - Soms
    - Zelden
    - Nooit

29. Duid aan hoe moeilijk of makkelijk de volgende zaken voor u zijn:

	Zeer moeilijk	Moeilijk	Niet moeilijk maar ook niet makke-lijk	Makke-lijk	Zeer makke-lijk
Na afloop van een bezoek aan uw arts extra informatie of duiding opzoeken over datgene dat uw arts met u besproken heeft.					
Administratieve zaken over uw gezondheid bijhouden (bv. afspraken met artsen of andere zorgverleners).					

Vraag 30-34: Deze vragen informeren naar uw huidige ervaringen met uw persoonlijk patiëntendossier

30. Duid aan in hoeverre u akkoord of niet akkoord gaat met de volgende stellingen:

	Hele-maal akkoord	Akkoord	Neutraal	Niet akkoord	Hele-maal niet akkoord
Ik vraag me weleens af wat er in mijn persoonlijk patiëntendossier staat					
Ik heb weleens de indruk gehad dat de inhoud van mijn persoonlijk patiëntendossier niet klopt					
Ik heb wel eens de indruk gehad dat er gegevens in mijn persoonlijk patiëntendossier staan waarvan ik niet op de hoogte was					
Het is mij duidelijk wie er toegang heeft tot de gegevens in mijn persoonlijk patiëntendossier					
Het is mij duidelijk wat er gebeurt met de gegevens in mijn persoonlijk patiëntendossier					
Als het mij gevraagd zou worden zou ik ermee akkoord gaan dat de gegevens in mijn persoonlijke patiëntendossier (geanonimiseerd) gebruikt worden voor onderzoek door wetenschappelijke instellingen zoals universiteiten					
Als het mij gevraagd zou worden zou ik ermee akkoord gaan dat de gegevens in mijn persoonlijke patiëntendossier (geanonimiseerd) gebruikt worden voor onderzoek door commerciële instellingen (bv. farmaceutische bedrijven, verzekeraars, ...)					

31. Duid aan hoe bezorgd u bent over de volgende zaken

	Zeer bezorgd	Bezorgd	Niet erg bezorgd	Niet bezorgd	Hele-maal niet bezorgd
Dat er mensen zijn die toegang hebben tot de gegevens in mijn persoonlijke patiëntendossier van wie ik liever had dat ze geen toegang hebben					
Dat de gegevens in mijn persoonlijke patiëntendossier ongeoorloofd gebruikt en verspreid kunnen worden					
Dat de gegevens in mijn persoonlijke patiëntendossier gebruikt kunnen worden voor commerciële doeleinden					

Dat mijn verzekering/mutualiteit mij anders zou behandelen als deze toegang zou hebben tot de gegevens in mijn persoonlijke patiëntendossier					
Dat mijn verzekering/mutualiteit mij zou uitsluiten van verzekeringen als deze toegang zou hebben tot de gegevens in mijn persoonlijke patiëntendossier					

32. Heeft u weleens toegang gevraagd tot uw persoonlijk patiëntendossier via een arts of andere zorgverlener?
    - o Ja
    - o Nee
    - o Weet ik niet

Vraag 33 is alleen zichtbaar voor deelnemers die 'ja' hebben geantwoord op vraag 32.

33. Duid aan in hoeverre u akkoord of niet akkoord bent met de volgende stelling: Ik kreeg gemakkelijk toegang tot mijn persoonlijk patiëntendossier
    - o Helemaal akkoord
    - o Akkoord
    - o Neutraal
    - o Niet akkoord
    - o Helemaal niet akkoord

34. Hoe belangrijk is het voor u om zelf te kunnen beslissen wie er toegang heeft tot de gegevens in uw persoonlijk patiëntendossier?
    - o Zeer belangrijk
    - o Belangrijk
    - o Niet zo belangrijk
    - o Onbelangrijk
    - o Zeer onbelangrijk

Vraag 35-29: Deze vragen informeren naar uw uw wensen in het geval u op een eenvoudige wijze toegang zou hebben tot alle gegevens in uw persoonlijk patiëntendossier

35. Duid aan hoe belangrijk zou u het vinden om de volgende zaken te kunnen doen als u zelf toegang zou hebben tot alle gegevens in uw persoonlijk patiëntendossier

	Zeer belang-rijk	Belang-rijk	Niet erg belang-rijk	Onbe-langrijk	Zeer onbe-langrijk
Recente persoonlijke gegevens vergelijken met persoonlijke gegevens uit het verleden					
Uw gegevens vergelijken met (medische) standaarden					
Uw gegevens vergelijken met de (geanonimiseerde) gegevens van andere patiënten					

Uw gegevens vergelijken met de (geanonimiseerde) gegevens van de Vlaamse populatie					
Verbanden bekijken tussen uw symptomen, uw aandoening(en), uw lichamelijke waarden, etc.					
Verbanden bekijken tussen uw gezondheid en de aanwezigheid van omgevingsfactoren					
De evolutie van uw gezondheid in functie van tijd bekijken					
Informatie bekijken over het verwachte effect van behandelingen op uw persoonlijke gezondheid					
Informatie bekijken over het verwachte effect van uw leefstijl op mijn persoonlijke gezondheid					
Uw gegevens (geanonimiseerd) beschikbaar stellen zodat regionale of landelijke problemen gedetecteerd kunnen worden					
Op de hoogte gebracht worden als bepaalde lichamelijke waarden evolueren richting gevaarlijke niveaus					
Op de hoogte gebracht worden als uw gezondheid verandert					

36. Als u toegang zou hebben tot alle gegevens in uw persoonlijk patiëntendossier, hoe moeilijk of makkelijk denkt u dat het zou zijn om zelf uw te vergelijken met (geanonimiseerde) gegevens van anderen?
    - Zeer moeilijk
    - Moeilijk
    - Niet moeilijk maar ook niet makkelijk
    - Makkelijk
    - Zeer makkelijk

37. Duid aan in hoeverre u akkoord of niet akkoord bent met de volgende stellingen:

	Hele-maal akkoord	Akkoord	Neu-traal	Niet akkoord	Hele-maal niet akkoord
Als ik de gegevens in mijn persoonlijke patiëntendossier zelf zou kunnen vergelijken met (geanonimiseerde) gegevens van anderen, dan maak ik vast fouten waardoor ik verkeerde conclusies trek					
Als ik toegang zou hebben tot alle gegevens in mijn persoonlijke patiëntendossier dan zou ik me onnodig zorgen gaan maken					
