# Supplementary material for: Designing a Patient Portal for Patient-Centered Care: Cross-Sectional Survey
Source: J Med Internet Res. 2018 Oct 1;20(10):e269. doi: 10.2196/jmir.9497 (PMC6231862; doi:10.2196/jmir.9497)
Supplement: Multimedia Appendix 3 [file jmir_v20i10e269_app3.pdf]

Multimedia Appendix 2: The Checklist for Reporting Results of Internet E-Surveys (CHERRIES).

MyHealthData Survey

Survey design
Convenience sample

IRB approval
This study was approved on 14 July 2015 (first part of the survey) by the Ethical Committee of the Faculty of Social Sciences of KU Leuven (SMEC) and on 24 September 2015 (second part of the survey) by the SMEC with number G-2015 07 272.

Informed consent
The first page of the survey contained the informed consent, with a mandatory checkbox for the participants to acknowledge they have read and agree with the informed consent. The final page of the survey contained a mandatory checkbox for participants to acknowledge they want to send the information to the researchers. If not, the data was not stored.

Data protection
The Google Forms and the Qualtrics platform were used for this survey, no additional measures regarding data protection were taken. Considering data protection, no personal identifiers were asked from the participants (Name, email). Participants could also participate on paper. Completed surveys were never coupled with the initial expression of interest, telephone number, nor postal addresses from the participant.

Development and testing
The survey was developed by general practitioners and user experience & usability experts. Once the survey was set up, it was piloted by 6 people and checked for usability improvements before distributing it.

Open survey versus closed survey
Open survey

Contact mode
The call for participation was spread both online and offline, both allowing web-based and paper-based data entry. In order to receive a paper copy of the survey, participants could call a dedicated telephone number that was only used to collect the participants' addresses on an answering machine, after which they received a paper copy including return envelope. The addresses were erased from the answering machine and never coupled with returned forms.

Advertising the survey
Inhabitants of the Flemish part of Belgium were invited to participate through several channels: the member magazine of the biggest Flemish health insurance; the online newsletter of the same health insurance; the newsletter of a website called 'health and

science' which is an independent and evidence-based website to inform patients about health-related topics; the website of a well-known Flemish weekly magazine (knack.be) and their social media channels (Facebook and Twitter). In addition, students from the Faculty of Social Sciences used their social networks to recruit participants. Two reminders were sent out by the health insurance and the 'Health and Science' newsletter to obtain the greatest possible response. People without access to a computer or the internet could phone to request a paper copy of the survey.

Call (translated from Dutch):

> Nowadays, all kinds of information regarding our health is gathered. Often, we don't have access to this data ourselves, but that is likely to change in the future. Think with us how this future could look like!
>
> In the scope of the research project MyHealthData (by KU Leuven and iMinds) we are looking for participants to complete our survey. Anyone can participate in the study. The survey asks about your experiences with medical care, the exchange of medical data between doctors and patients, your opinion on patient records, ... Next to that, the survey includes some questions about what exactly you would like to know about your own health.
>
> More information and the survey via: www.bit.ly/vragenlijststudie. In case you don't have access to the internet, you can call the following telephone number to request a paper version of the survey: 016/37 41 11

Survey Administration: Web/E-mail
The main channel was through the Google Forms platform. A secondary channel was through the Qualtrics platform (used by the students from the Faculty of Social Sciences). Participants could enter a paper version of the survey, which were entered manually in the spreadsheet with entries. Digital entries and manually entered entries from the paper surveys were marked in an additional column.

Context
Primary a dedicated Google Forms webpage was used, in combination with a shortened URL for easy access (bit.ly). Secondary the Qualtrics platform was used by students. The informed consent, layout and content of the survey were identical for both platforms.

Mandatory/Voluntary
Voluntary, participants could stop at any moment. The data was only stored after the participants completed the full survey and the mandatory checkbox was checked at the final page of the survey.

Incentives
No

Time/Date

The data was collected between 25 March and 1 September 2016.

Randomization of items
No

Adaptive questioning
Yes, in the first section after the informed consent, participants had to answer whether they have a chronic condition. If not, all following questions that related to chronic conditions were skipped.

Number of items
Excluding the informed consent at the start and final check at the end, the survey contained 152 items spread over 16 sections.

Number of screens
Eighteen screens, including one opening screen for the informed consent and one concluding screen for the final consent.

Review step
Through Back/Next buttons, participants could review and change their responses.

Unique site visitor
We could not determine unique visitors. To protect participants' identities, it was decided not to require participants to login using their email address—the mechanism Google Forms uses to identify participants and guarantee unique visitors.

View rate
We did not determine unique visitors, so we can't provide view rates.
The Google Forms platform received 709 clicks which resulted in 349 submitted surveys. The use of the Qualtrics platform resulted in 74 submitted surveys.

Participant rate
We did not determine unique visitors, so we can't provide participant rates.

Completion rate
Online we received 423 completed informed consent pages and there were 37 surveys where the last question was not answered, so our completion rate was 91%.
From the 11 paper surveys that were requested and sent to participants, 10 completed surveys were received (all fully completed).

Preventing multiple entries from the same individual
Standard Google form functionality was used. It was decided not to use the Google Forms mechanism to prevent multiple entries from the same individual as it requires participants to log in using their email address. The Qualtrics survey protection option to keep people from taking the survey more than once was turned on.

Handling of incomplete questionnaires

Online questionnaires where participants gave their final consent were stored. So also incomplete questionnaires could be submitted if the final checkbox was checked. If we found blank surveys which only had their 2 checkboxes completed but not a single question answered, we did not use them. All received paper surveys were fully completed.

Questionnaires submitted with an atypical timestamp
Not taken into account

Statistical correction
To calculate the frequencies, missing data were excluded and percentages were based on the number of non-missing values.
For the linear regression, only 1 independent variable had more than 10% missing data (age, 13.2%). All other independent variables had less than 10% missing data so we didn't impute data. If values of any of the independent variables included missing data, the entire case was excluded for the analysis. For the dependent variables we used the mean sum score to compensate for missing data.
