# Supplementary material for: Designing a Patient Portal for Patient-Centered Care: Cross-Sectional Survey
Source: J Med Internet Res. 2018 Oct 1;20(10):e269. doi: 10.2196/jmir.9497 (PMC6231862; doi:10.2196/jmir.9497)
Supplement: Multimedia Appendix 4 [file jmir_v20i10e269_app4.pdf]

Multimedia Appendix 4: Qualitative Study Leading to Part 2 of the survey:

The MyHealthData project investigated how patient information could be disclosed to patients in the Flemish population. Following a Human-Centered Design process [1] end-users were involved early in the design process to study and understand their actual needs before defining any functionality of the Patient Portal (PP) and designing it.

In a qualitative study, we aimed to understand why people are interested in their own personal health information, what current experiences they have in retrieving relevant health information, and why they would be interested in a PP. As no PP was being used in Flanders, the target population had no experience with it at all. As Sanders' experience model [2] points out, expectations towards the future are based on memories, making it difficult to express expectations towards non-familiar technologies, such as a PP for the Flemish population in this study. Having no experience with the concept of a PP, people have difficulties imagining such a system or how it could be useful to them, let alone expressing this. Based on Visser, Stappers, van der Lugt and Sanders [2], a generative approach was taken to overcome this difficulty.

The qualitative study consisted out of 3 steps: we asked people to make a set of assignments at home, then invited them for a focus group in our lab or interview at their home, and concluded the study with a questionnaire validating the outcomes of the preceding steps with a larger population. Based on a literature review, 3 groups of participants were selected who are expected to have a high interest in health information from 3 different perspectives: expecting couples, people who were treated for cancer, and people with a chronic condition.

First, the recruited participants received a set of assignments to complete on their own at home. The assignments, a set of cultural probes [3] were specifically designed for this study. Recruited participants received a box with the cultural probes at home by mail. When opening the box, the participant found a sheet with instructions and an informed consent. We asked the participants to complete the assignments by the next encounter: the focus group or individual interview that was scheduled two weeks later. The box contained seven assignments (see figures 1 and 2): 1. a personal profile or ID telling us who they are and what they value; 2. a timeline giving an overview of both personal and medical events during their lifetime the health related questions that emerged then; 3. a letter we asked them to write to a fellow-sufferer, containing advice they would want to give; 4. a mind-map collecting health related thoughts and questions in specific situations such as during travel or at work; 5. a 'yellow pages' booklet to note all their current sources for health information; 6. a drop box to collect any loose idea or thought related to health information during the two-week time period, this probe was targeted at the household rather than the participant alone; and 7. a photo album to indicate which snapshots of health information that is stored somewhere right now they would like to collect, for example X-rays.

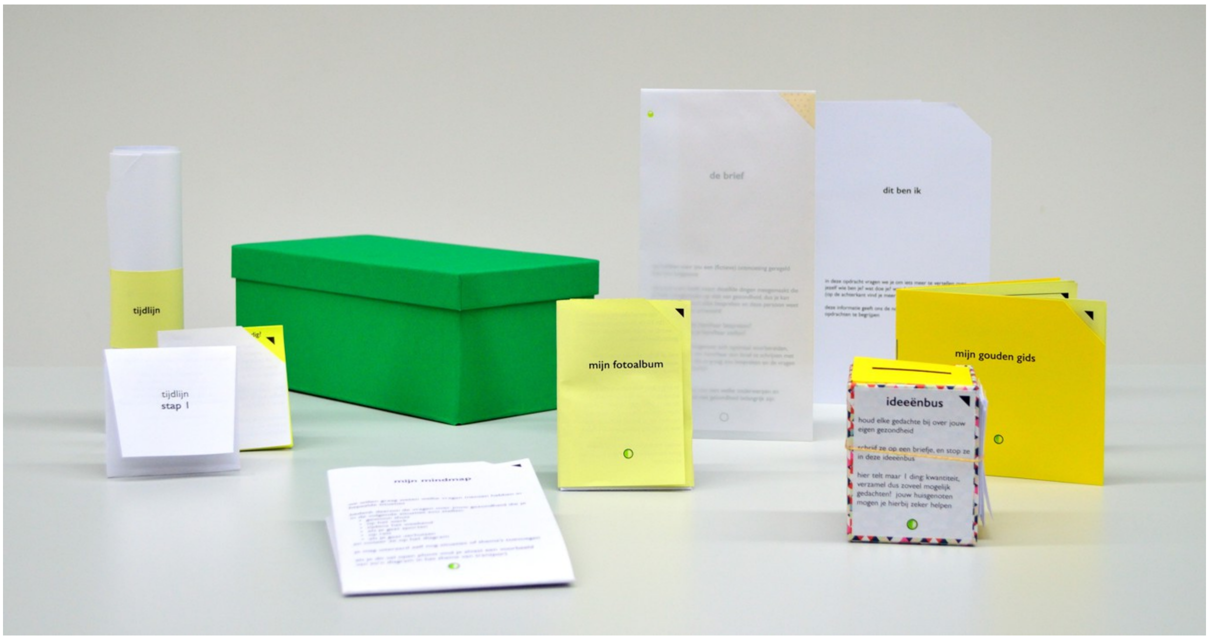

Figure 1: a cultural probes box with seven assignments, from left to right: timeline with stickers and post-its, the box, folded mindmap, photo album, letter in envelope, profile, dropbox with paper snippets, yellow pages booklet.

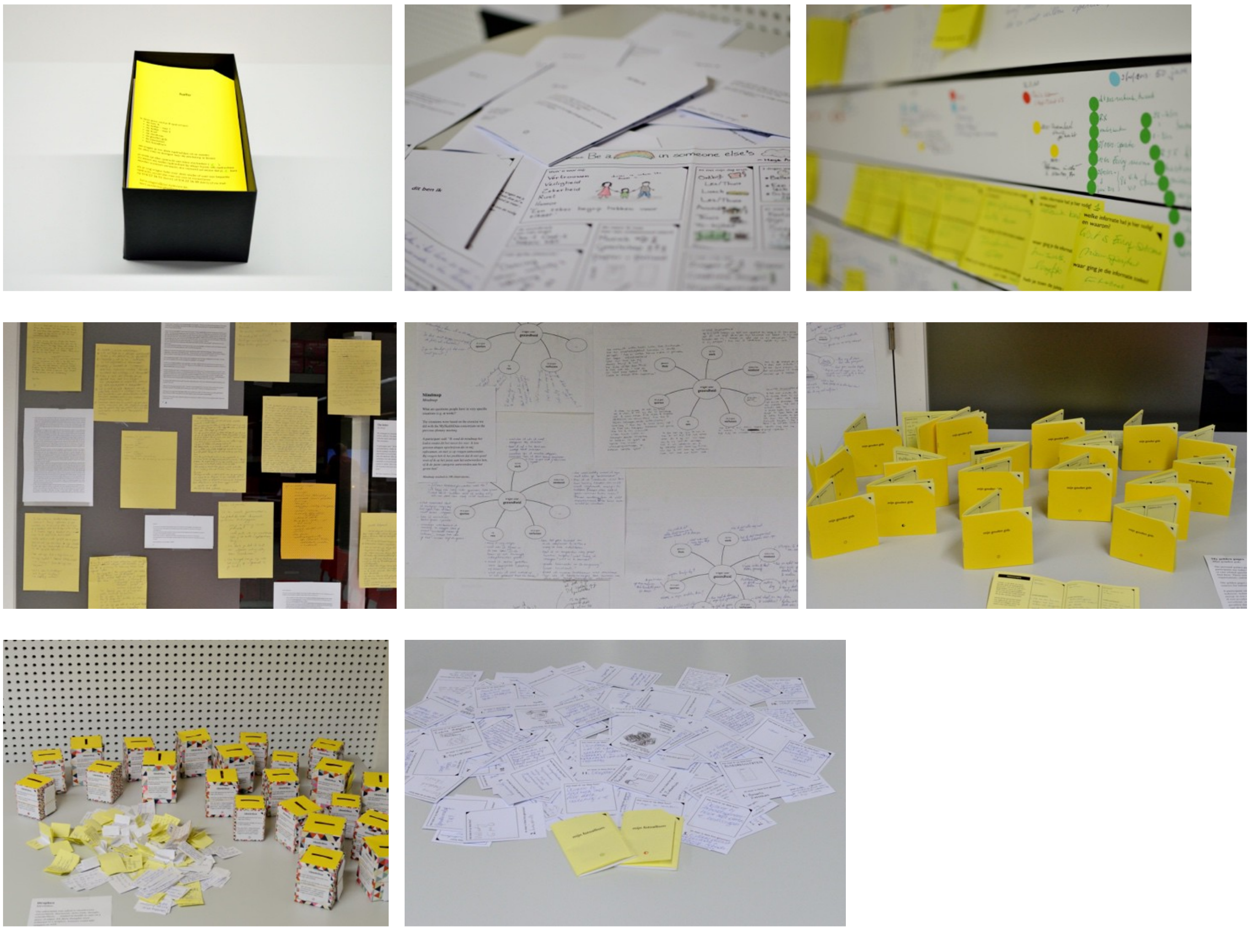

Figure 2: completed probes: opened box with instructions, profile, timeline, letters, mindmaps, yellow pages, droboxes with paper snippets, collected photos in photo albums.

In a second phase, the participants were invited to a focus group at the research lab to join a brainstorm on PP functionality. As multiple participants had difficulties traveling to the research lab and attending the focus groups due to their medical conditions, we also visited participants at home for an in-depth interview discussing the assignments and a similar brainstorm as in the focus groups. In total, 9 participants joined one of the 3 focus groups (4 chronic patients, 2 pregnant parents, 3 people who finished cancer treatment) and 14 participants were interviewed at home (6 chronic patients, 4 pregnant or new parents, 4 people who finished cancer treatment). As health information is not a topic which is on top of mind for most people, the assignments of the previous step helped participants to be prepared to think about PP functionality in the brainstorm. During the focus group and after the individual interviews, participants generated a list of ideas regarding PP functionalities: what would they find interesting, why would this be interesting to them, and how would they expect this functionality to be implemented? The method we used in this focus group was a step-by-step process relying on a metaphor for the PP, abstracting the technology from people's interests and needs. [4]

In a third and final step, the researchers analyzed the received inputs of the assignments and focus groups, which lead to a questionnaire to be completed in a quantitative study (part 2 of the reported questionnaire in this article). All individual interviews were transcribed, and all completed assignments were entered in a spreadsheet. This input was then analyzed by two researchers following grounded theory, through open, axial, and selective coding. Seven themes emerged from the research data: 1: health awareness; 2: coping; 3: effective care; 4: empowerment; 5: good health; 6: patient rights; 7: getting recognition. These themes grouped several items, such as that people want to understand whether and when care is necessary and compare possible treatments (effective care) or what reimbursements they are entitled to (patient rights). To validate these findings with a larger population a questionnaire was constructed based on these items. Combining part 1 and 2 of the questionnaire allowed us to compare a quantitative research instrument with our bottom-up qualitative research method. For creating part 2 of the questionnaire, the subthemes that were identified for each of the seven themes listed above were translated to a neutral question about, for instance, the importance of certain health information, ("How important is it for you…"), concerns people may have ("To what extent are you concerned about …"), the difficulty people experience with health care ("How easy or difficult is it for you to…"), people's health-related behavior ("Before I visit my doctor, I try to find relevant information to be well-prepared."), etc. Questions could be answered by means of a five-point Likert scale (ranging from very important to very unimportant, from very concerned to not concerned at all, from very easy to very difficult, or from always to never). The questions were formulated as neutral as possible to prevent any bias or steering in certain directions.

References

1. URL:https://www.iso.org/standard/52075.html. Accessed: 2018-04-18. (Archived by WebCite® at http://www.webcitation.org/6ylzUJ8MP)
2. Visser FS, Stappers PJ, van der Lugt R, Sanders EBN. Contextmapping: experiences from practice. CoDesign. 2005 2005/04/01;1(2):119-49. doi: 10.1080/15710880500135987.
3. Gaver B, Dunne T, Pacenti E. Design: cultural probes. interactions. 1999;6(1):21-9.

4. Vandenberghe B, Slegers K, editors. Anthropomorphism as a Strategy to Engage End-Users in Health Data Ideation. Proceedings of the 9th Nordic Conference on Human-Computer Interaction; 2016: ACM.
