# Supplementary material for: Designing a Patient Portal for Patient-Centered Care: Cross-Sectional Survey
Source: J Med Internet Res. 2018 Oct 1;20(10):e269. doi: 10.2196/jmir.9497 (PMC6231862; doi:10.2196/jmir.9497)
Supplement: Multimedia Appendix 5 [file jmir_v20i10e269_app5.pdf]

Multimedia Appendix 5: predictor variables: Demographic variables, patient-empowerment variables and health literacy variables

Predictors	Variable choices	Condensed values
**Age**	Free entry	18 - 34 years
		35 - 44 years
		45 - 54 years
		55 - 64 years
		65 and older (65+)
**Employment Status**	Employed full of part time	Working
	Self - employed	Student
	Full time student	Not working
	Homemaker	Retired
	Disabled and not working	
	Curently unemployed, not working	
	Retired	
**Family income**	Under 20 000 €	<30 000 €
	20 000 - 30 000 €	30 000 - 60 000 €
	30 000 - 40 000 €	>60 000 €
	40 000 - 60 000 €	Don't know / don't wish to answer
	60 000 - 80 000 €	
	Above 80 000 €	
	Don't know	
	Don't wish to answer this question	
**Health status**	Excellent	Excellent
	Very good	Good/fair
	Good	Poor
	Fair	
	Poor	
**Education**	Primary school	Highschool or lower
	High school	Bachelor degree
	Bachelor degree	Master degree or higher
	Master degree or higher	
**Gender**	Male	Male
	Female	female
**Shared decision making** [a]	Strongly agree	Agree
	Agree	Neutral

	Neutral	Disagree
	Disagree	
	Strongly disagree	
**Questioning the decisions of physicians** [a]	Very important	Important
	Important	Neutral
	Not very important	Not important
	Unimportant	
	Very unimportant	
**Satisfaction with healthcare last 5 years** [a]	Very satisfied	Satisfied
	Somewhat satisfied	Neutral
	Neutral	Dissatisfied
	Somewhat dissatisfied	
	Very dissatisfied	
**Finding relevant health information** [b]	Very difficult	Difficult
	Difficult	Not easy /not difficult
	Not easy / not difficult	Easy
	Easy	
	Very easy	
**Evaluating the reliability of health information** [b]	Very difficult	Difficult
	Difficult	Not easy /not difficult
	Not easy / not difficult	Easy
	Easy	
	Very easy	
**Problems understanding health information** [b]	Always	Often
	Often	Sometimes
	Sometimes	Rarely
	Occasionally	
	Never	

a) Used as variables for patient-empowerment

b) Used as variables for health literacy
